# Leptin Promotes Wound Healing in the Oral Mucosa

**DOI:** 10.1371/journal.pone.0101984

**Published:** 2014-07-17

**Authors:** Hirochika Umeki, Reiko Tokuyama, Shinji Ide, Mitsuru Okubo, Susumu Tadokoro, Mitsuki Tezuka, Seiko Tatehara, Kazuhito Satomura

**Affiliations:** Department of Oral Medicine and Stomatology, Tsurumi University School of Dental Medicine, Yokohama, Kanagawa, Japan; National Centre for Scientific Research, ‘Demokritos’, Greece

## Abstract

**Introduction:**

Leptin, a 16 kDa circulating anti-obesity hormone, exhibits many physiological properties. Recently, leptin was isolated from saliva; however, its function in the oral cavity is still unclear. In this study, we investigated the physiological role of leptin in the oral cavity by focusing on its effect on wound healing in the oral mucosa.

**Methods:**

Immunohistochemical analysis was used to examine the expression of the leptin receptor (Ob-R) in human/rabbit oral mucosa. To investigate the effect of leptin on wound healing in the oral mucosa, chemical wounds were created in rabbit oral mucosa, and leptin was topically administered to the wound. The process of wound repair was histologically observed and quantitatively analyzed by measuring the area of ulceration and the duration required for complete healing. The effect of leptin on the proliferation, differentiation and migration of human oral mucosal epithelial cells (RT7 cells) was investigated using crystal violet staining, reverse transcription polymerase chain reaction (RT-PCR) and a wound healing assay, respectively.

**Results:**

Ob-R was expressed in spinous/granular cells in the epithelial tissue and vascular endothelial cells in the subepithelial connective tissue of the oral mucosa. Topical administration of leptin significantly promoted wound healing and shortened the duration required for complete healing. Histological analysis of gingival tissue beneath the ulceration showed a denser distribution of blood vessels in the leptin-treated group. Although the proliferation and differentiation of RT7 cells were not affected by leptin, the migration of these cells was accelerated in the presence of leptin.

**Conclusion:**

Topically administered leptin was shown to promote wound healing in the oral mucosa by accelerating epithelial cell migration and enhancing angiogenesis around the wounded area. These results strongly suggest that topical administration of leptin may be useful as a treatment to promote wound healing in the oral mucosa.

## Introduction

The oral cavity is the entry point for the alimentary and respiratory tracts. The surface of the oral cavity is covered by oral mucosa, a moist lining that communicates with the external environment. The oral mucosa consists of two separate tissue components: stratified squamous epithelium (the oral epithelium) and an underlying connective tissue layer (the lamina propria). Functions of the oral mucosa include protection, sensation and secretion [1]. The oral mucosa protects deeper tissues and organs in the oral region by separating them from the external environment. There is normally a resident population of microorganisms within the oral cavity that could cause infection if they gained access to the tissues. The oral mucosa, particularly the oral epithelium, is the major barrier to these exogenous threats [2]. Numerous minor salivary glands are also associated with the oral mucosa, and the saliva secreted by these glands contributes moistening, lubrication [3] and topophylaxis within the oral cavity.

The oral mucosa is also sensitive to a variety of stresses: physiological stress such as mechanical stimuli from ill-fitting prostheses and heat from food or beverages; chemical irritation from tobacco smoking; and biological stress from resident microorganisms. Maintaining the integrity of the oral mucosa is critically important not only for oral function but also for general health.

Leptin, a 16-kDa non-glycosylated polypeptide anti-obesity hormone consisting of 146 amino acids, is a product of the *obese* (*ob*) gene [4]. Although leptin is mainly produced by white adipose tissue [4], recent studies have demonstrated that leptin is also produced by placenta [5], stomach [6], skeletal muscles [7], brain and pituitary gland [8], [9]. Leptin is known to exhibit a variety of physiological actions on body weight homeostasis [10], lipid metabolism [11], hematopoiesis [12], thermogenesis [13], ovarian function [14], bone formation [15], [16], angiogenesis [17], [18] and wound healing [19], [20], [21]. The leptin receptor (Ob-R) is expressed in various tissues including the hypothalamus [22], [23], adipose tissue [24], skeletal muscle [25] and hepatocytes [24], [26]. The multifunctionality of leptin and the wide distribution of its receptor suggest that leptin plays a variety of physiological roles not only as a systemic hormone but also as a local growth factor. A past study found that leptin exists in human saliva as well as serum [27], [28]. However, the physiological role of leptin in the oral cavity remains to be elucidated. In the present study, we investigated the physiological role of leptin in wound healing in the oral mucosa.

## Materials and Methods

### Human tissue samples

The experimental protocol of this study was approved by the Research Ethics Review Committee of Tsurumi University School of Dental Medicine (approval number 1048). After written informed consent was obtained, small samples of buccal mucosa were obtained from two healthy volunteers (25 to 30 years of age) at Tsurumi University Dental Hospital using a disposable dermal punch (5 mm in diameter; Nipro, Osaka, Japan) under local anesthesia, and the wound was surgically sutured. The tissues were fixed with 10 N Mildform (Wako Pure Chemical Industries Ltd, Osaka, Japan) and embedded in paraffin. Sections were cut at 5 µm thickness, deparaffinized and stained with hematoxylin and eosin.

### Creation of chemical wounds

The animal care and experimental protocols were approved by the Committee on the Ethics of Animal Experiments of Tsurumi University (Permit Number: 25A042). All surgery was performed under sodium pentobarbital anesthesia, and all efforts were made to minimize suffering. Twenty-four Japanese white rabbits (2.5–3.0 kg, male) were obtained from Tokyo Laboratory Animals Science Co., Ltd (Tokyo, Japan), fed a normal diet and maintained under a 12-hour-light/12-hour-dark cycle at 22°C. Chemical wounds were created on the rabbit oral mucosa by applying a piece of filter paper (*φ*5 mm) soaked with 50% acetic acid for 2 minutes to the mandibular gingiva. Wound formation was verified after 1 day, and a 10 µl of 100 ng/ml leptin or phosphate-buffered saline (PBS) (as a control) was mixed with 90 µl of Cellmatrix (type I collagen, Nitta Gelatin Inc., Osaka, Japan) and topically applied to the wound daily. The ulcer size was measured on day 6 and day 13 after wound formation, and the gingival tissues around the wound were obtained for histological analysis. Excised tissue was fixed with 10 N Mildform (Wako) and embedded in paraffin. Sections were cut at 5 µm thickness, deparaffinized and stained with hematoxylin and eosin. In addition, body weight (BW), and aspartate aminotransferase (AST), alanine amino transferase (ALT), and blood sugar (BS) levels were also measured to detect adverse effects.

### Immunohistochemistry

Sections of human buccal mucosa and rabbit gingiva were transferred onto poly-l-lysine-coated glass slides (Matsunami Glass, Osaka, Japan). After deparaffinization with xylene and rehydration with descending concentrations of ethanol, endogenous peroxidase was blocked by treatment with 3% H_2_O_2_ in methanol for 1 hour at room temperature (RT). After treatment with 10% normal rabbit serum at RT for 10 min, sections were incubated with the primary antibodies (goat polyclonal antibody against Ob-R, Santa Cruz Biotechnology, Inc., CA, USA) diluted 1∶500 in PBS (pH 7.4) containing 1% bovine serum albumin at 4°C overnight. After washing with PBS, the localization of Ob-R was visualized using a Histofine SAB-PO (G) kit (Nichirei Corporation, Tokyo, Japan) and a 3,3′-diaminobenzidine (DAB) substrate kit (Nichirei). Sections were counterstained with hematoxylin and mounted. The specificity of the immunoreaction was confirmed by incubation with normal goat IgG and normal goat serum instead of the primary antibodies.

### Histometric analysis

A histometric analysis was performed using three tissue sections at day 6 after wound creation. Twenty fields (750 µm×500 µm each) in the subepithelial connective tissue beneath the ulcer were arbitrarily selected and the blood vessels were counted.

### Cell culture

RT7 cells, an oral mucosal epithelial cell line derived from human buccal mucosa [29], was a generous gift from Dr. Nobuyuki Kamata, Hiroshima University, Hiroshima, Japan. RT7 cells were cultured in KGM-Gold Basal Medium (Lonza Ltd, Basel, Switzerland) supplemented with 0.4% bovine pituitary extract (BPE), 0.1% recombinant human epidermal growth factor (rhEGF), 0.1% bovine insulin, 0.1% hydrocortisone, 0.1% transferrin, 0.05% epinephrine, 0.1% gentamicin and 0.1% amphotericin-B. The culture was maintained at 37°C in a humidified atmosphere of 5% CO_2_ in air and the medium was changed twice a week.

### Cell proliferation assay

The effect of leptin on the proliferation of RT7 cells was analyzed using a crystal violet staining method [30]. In brief, cells were plated at a cell density of 4×10^3^ cells per well in 12-well culture plates. Cells were treated with various concentrations (0, 0.1, 1, 10 ng/ml) of leptin for 1, 3, 5, 7, 10, 14, 17 and 20 days. On each scheduled day, cells were rinsed with PBS and fixed with 1% glutaraldehyde in PBS overnight at 4°C. The cells were then stained with 0.02% crystal violet in deionized water for 30 min at RT. After several rinses with distilled water, crystal violet bound to cells was extracted by overnight incubation with 500 µl/well of 70% ethanol at 4°C. Absorbance was measured at 570 nm using a microplate reader Model 680 (Bio-Rad, California, USA).

### Semi-quantitative RT-PCR analysis

RT7 cells were seeded into 60 mm petri dishes at a cell density of 1×10^5^ cells/dish, and cultured until they reached confluence. The day at confluence was designated as day 0. We first confirmed the expression of Ob-R mRNA in RT7 cells by reverse transcription polymerase chain reaction (RT-PCR) analysis. Thereafter, RT7 cells were treated with various concentrations (0, 0.1, 1, 10 ng/ml) of leptin for various periods. The expression of mRNA encoding *Keratin 4*, *Keratin 10*, *Transglutaminase I* and *G3PDH* was examined by semi-quantitative RT-PCR analysis. In brief, on each scheduled day, total RNA was extracted from RT7 cells using TRIzol reagent (Invitrogen, Carlsbad, USA), and cDNA was generated from 1 µg of the total RNA using SuperScript III First-Strand Synthesis System (Invitrogen). The PCR amplification was carried out in a 50 µl reaction mixture using 1.1x ReddyMix PCR Master Mix (1.5 mM MgCl_2_: ABgene, Thermo Scientific, Waltham, USA). Conditions and primer sequences for PCR amplification are shown in Table 1. The *G3PDH* gene was used as an internal control for the quantity and quality of cDNA. The PCR products were analyzed by ethidium bromide staining after separation by electrophoresis through a 2% agarose gel.

**Table 1:** Oligonucleotide primers used in RT-PCR.

Primers		
(GeneBank accession no.)	Sequence	Size (bp)
G3PDH	F: 5′-ACC ACA GTC CAT GCC ATC AC-3′	452
(NM_002046.4)	R: 5′-TCC ACC ACC CTG TTG CTG TA-3′	
Keratin 4	F: 5′-AGG CTT TGG CAC TGG TGG CTT T-3′	316
(BC_042174)	R: 5′-CCA TTT GGT CTC CAG GAC CTT A-3′	
Keratin 10	F: 5′-CGT GGA AGC TAT GGA AGT AGC AGC-3′	328
(BC_034697)	R: 5′-CAC GAG GCT CCC CCT GAT GTG AG-3′	
Transglutaminase I	F: 5′-ATG GAT GGG CCA CGT TCC GAT-3′	281
(XM_05268029)	R: 5′-TCA GAG GAT TCA TAG GTC CGG-3′	
Ob-R	F: 5′-GCT ATT TTG GGA AGA TGT-3′	499
(NM_002303)	R: 5′-TGC CTG GGC CTC TAT CTC-3′	

*F:* forward, *R:* reverse.

### Real-time RT-PCR analysis

The expression of mRNA encoding *keratin 4*, *keratin10, Transglutaminase I* and *G3PDH* in RT7 cells was also examined by real-time PCR. PCR was performed with SYBR Premix Ex Taq (Takara Bio Inc., Shiga, Japan) using an Applied Biosystems StepOne Real-Time PCR System (Applied Biosystems Inc., Carlsbad, CA, USA). Conditions and primer sequences for PCR amplification are shown in Table 2. The *GAPDH* gene was used as an internal control for the quantity and quality of cDNA.

**Table 2:** Oligonucleotide primers used in real-time RT-PCR.

Primers		
(GeneBank accession no.)	Sequence	Size (bp)
G3PDH	F: 5′-GAG TCA ACG GAT TTG GTC GT-3′	248
(NM_002046.4)	R: 5′-TTGATTTTGGAGGGATCTCG-3′	
Keratin 4	F: 5′- GCAGCTAGATACCTTGGGCAA-3′	95
(NM_042174.1)	R: 5′-GTGGAGGACTTCAAGACTAAGTATGAAG-3′	
Keratin 10	F: 5′-AGC ATG GCA ACT CAC ATC AG-3′	126
(BC_034697.1)	R: 5′-TGT CGA TCT GAA GCA GGA TG-3′	
Transglutaminase I	F: 5′-CAT CAA GAA TGG CCT GGT CT-3′	110
(XM_005268029.1)	R: 5′-CAA TCT TGA AGC TGC CAT CA-3′	

*F:* forward, *R:* reverse.

### Wound healing assay

The effect of leptin on the migration of RT7 cells was analyzed with a CytoSelect Wound Healing Assay kit (Cell Biolabs Inc., San Diego, USA). The assay was performed according to the manufacturer’s instructions. In brief, RT7 cells were seeded into 24-well plates and cultured overnight. After the cells were treated with various concentrations (0, 0.1, 1, 10 ng/ml) of leptin, the inserts, which were equipped to make a 0.9 mm gap to simulate a wound, were removed from the wells to begin the wound healing assay. Each gap was measured at 0, 12, 24, and 36 hours after the removal of the inserts.

### Quantitative assay of epidermal growth factor (EGF) and keratinocyte growth factor (KGF)

To elucidate the effect of leptin on the expression of EGF and KGF in RT7 cells, quantitative analysis of 16-fold concentrated supernatant of RT7 cells cultured in the presence of various concentrations (0, 0.1, 1, 10 ng/ml) of leptin was performed. For assay, Quantikine ELISA kit for EGF (R&D Systems, Minneapolis, USA; linear range was 3.9–250 pg/ml, limit of detection was 0.7 pg/ml) and Quantikine ELISA kit for KGF (R&D; linear range was 31.2–2000 pg/ml, limit of detection was 15 pg/ml) were used according to the manufacturer’s instructions.

### Statistics

All data are expressed as the mean ± standard error (SE). Statistical analysis was performed using the Kruskal-Wallis *H* test. Values of P<0.05 were considered statistically significant.

## Results

### Expression of Ob-R in human and rabbit oral mucosa

**Figure 1:**
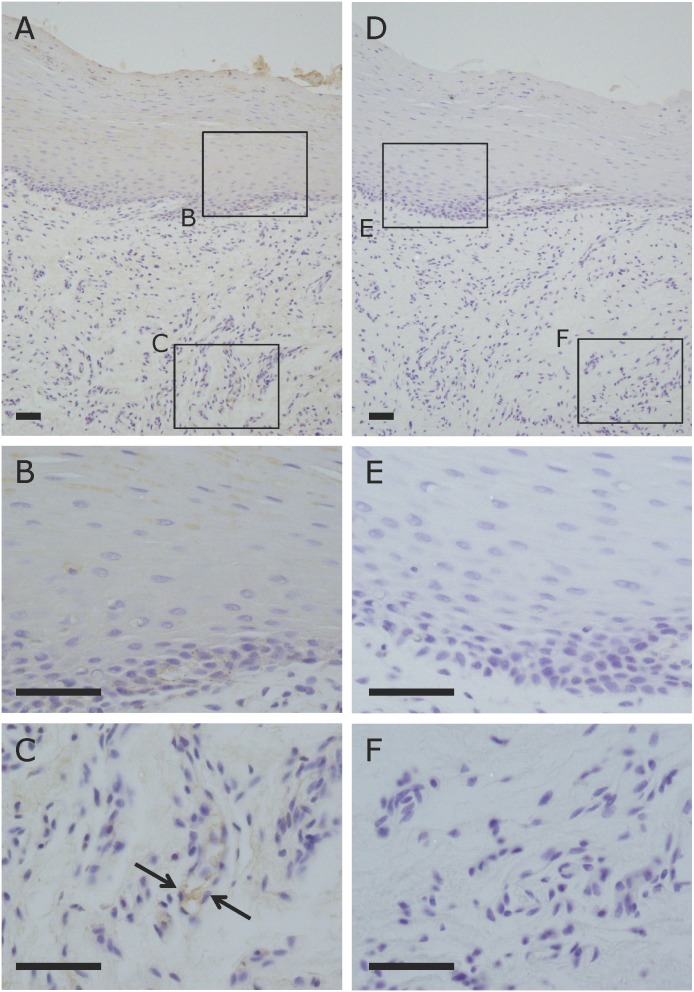
Immunohistochemical localization of Ob-R in human oral mucosa. (A–C) Immunohistochemical staining for Ob-R. DAB and hematoxylin staining. (B) Ob-R was expressed in spinous/granular cells of epithelial tissue. (C) Vascular endothelial cells (arrows) in subepithelial connective tissue were also positive for Ob-R. (D–F) Negative control. Scale bars = 50 µm.

Immunohistochemical analysis of the expression/localization of Ob-R in human oral mucosa revealed that Ob-R was expressed in the spinous and granular cells of epithelial tissue (Fig. 1A and 1B). The vascular endothelial cells in subepithelial connective tissue of the oral mucosa were also positive for Ob-R (Fig. 1A and 1C). Importantly, the expression pattern of Ob-R in oral mucosa was common between human and rabbit (data not shown).

### Effect of leptin on wound healing in the oral mucosa

**Figure 2:**
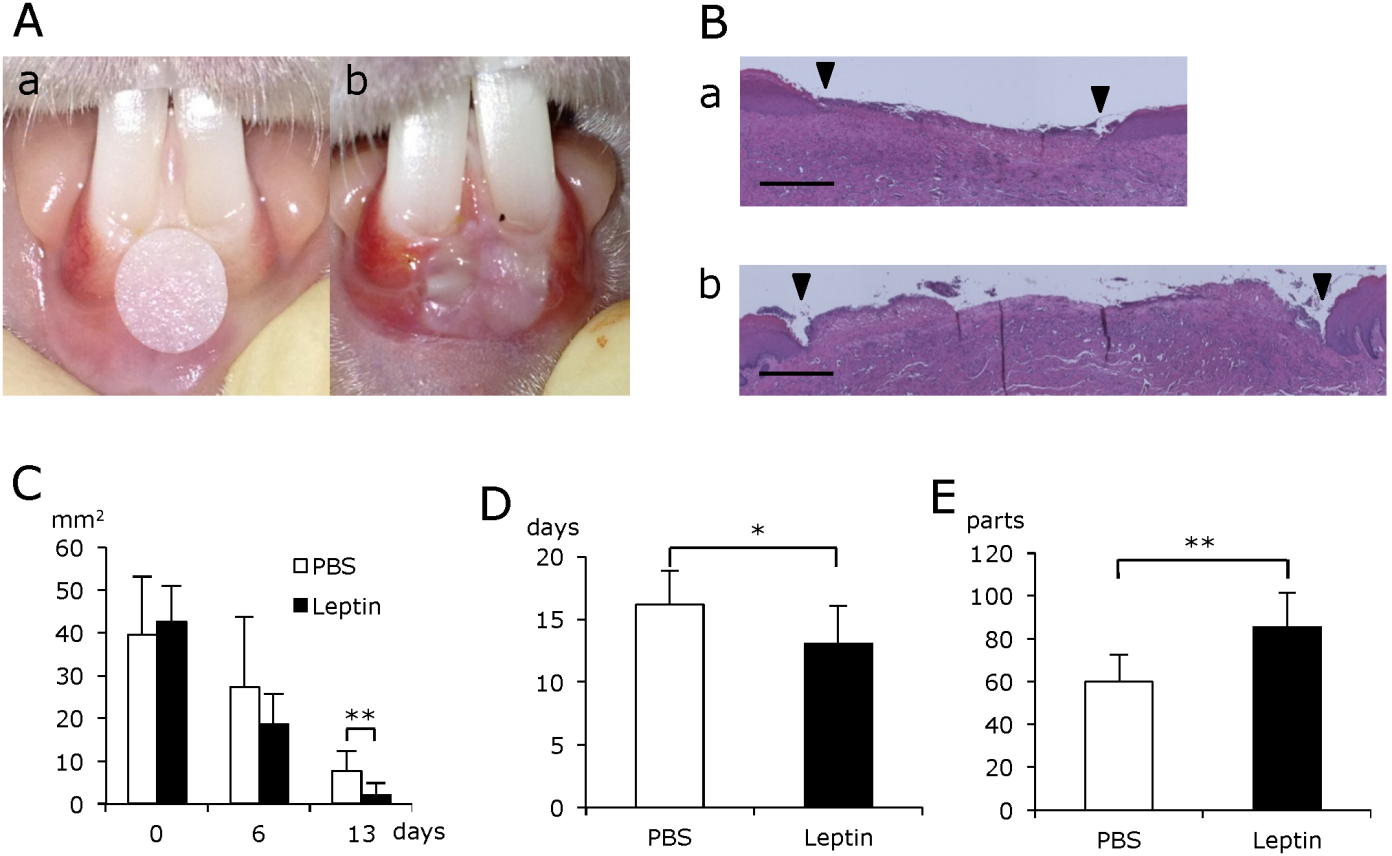
Effect of leptin on wound healing of oral mucosa. (A) Chemical wounds created in rabbit gingiva. (a) Filter paper (5 mm in diameter) soaked with 50% acetic acid attached to rabbit mandibular gingival for 2 minutes. (b) Gingival ulcer formed on the day following wound creation. (B) Histological findings during wound repair at day 6 after wound creation. Spaces between the two arrowheads show ulcerated area without epithelial lining. H-E staining. Scale bars = 500 µm. (C) Sequential changes in ulcerated area. (D) Total duration for complete healing of rabbit gingival ulcer. (E) Number of blood vessels in the subepithelial connective tissue beneath the ulcerated area. Values are mean ± SE from 12 animals per group. *P<0.05, **P<0.01.

**Figure 3:**
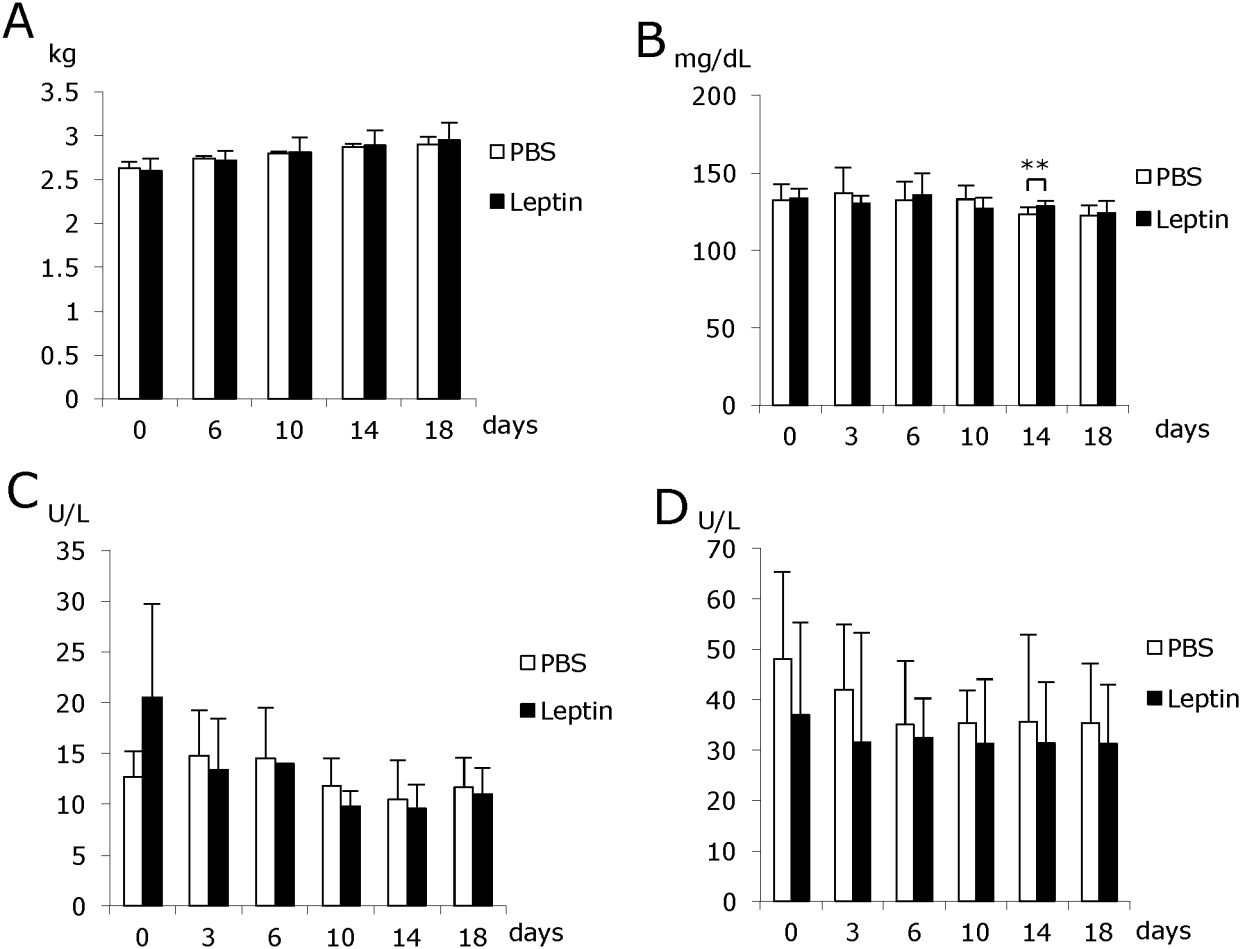
Changes in body weight (A), and serum levels of blood sugar (B), AST (C) and ALT (D) in the healing period. Generally, none of these laboratory parameters were significantly affected by leptin application throughout the experimental period. Values are mean ± SE from 5 animals per group. **P<0.01.

To elucidate the effect of topically administered leptin on wound healing in the oral mucosa, Cellmatrix containing leptin or PBS was directly applied to the gingival wounds (Fig. 2A). Thereafter, the size of the ulcer was measured sequentially in addition to histological examination. Histological observation showed that the wound area decreased much faster in the leptin-treated group compared with the control group (Fig. 2B and 2C). Moreover, the duration required for complete healing of the ulcer was shortened significantly in the leptin-treated group (Fig. 2D). Histometric analysis of the distribution of blood vessels revealed that more blood vessels were formed in the connective tissue beneath the ulcer in the leptin-treated group compared with the control group (Fig. 2E). Meanwhile, BW, and levels of AST, ALT or BS were not affected by leptin administration throughout the whole experimental period, which suggests that topically administered leptin caused no adverse effects (Fig. 3).

### Effect of leptin on human oral mucosal epithelial cells

**Figure 4:**
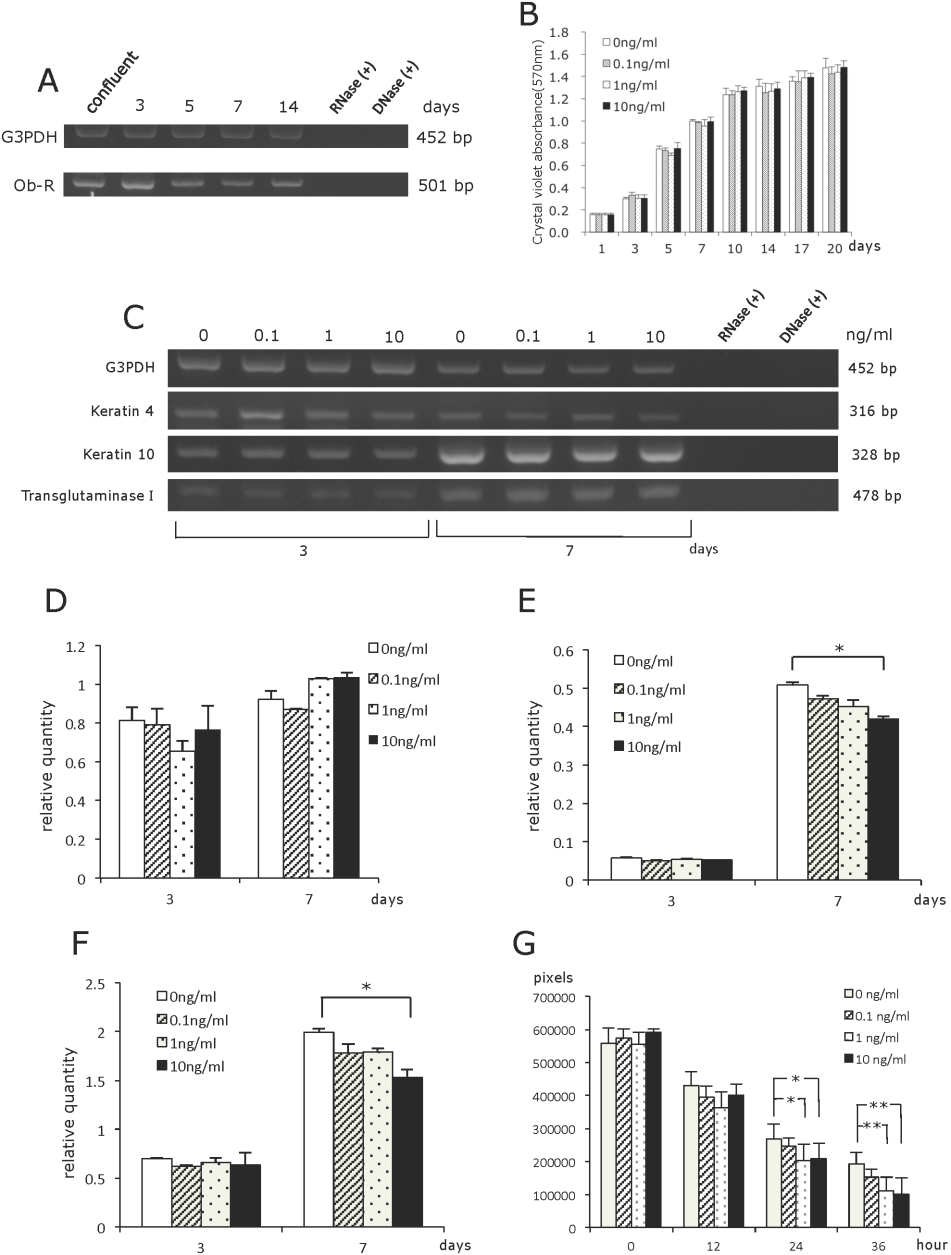
Effect of leptin on a human oral mucosal cell line (RT7 cells). (A) *Ob-R* mRNA expression in RT7 cells. RNase (+): Total RNA treated with RNase. DNase (+): cDNA treated with DNase. (B) Effect of leptin on the proliferation of RT7 cells. (C) Semi-quantitative RT-PCR analysis. Effect of leptin on the expression of mRNA encoding oral mucosal epithelial cell-related genes (*Keratin 4*, *Keratin 10* and *Transglutaminase I*) in RT7 cells. RNase (+): Total RNA treated with RNase. DNase (+): cDNA treated with DNase. (D) Real-time quantitative RT-PCR analysis of *Keratin 4*. Values are mean ± SE (n = 8). (E) Real-time quantitative RT-PCR analysis of *Keratin 10*. Values are mean ± SE (n = 8). *P<0.05 (F) Real-time quantitative RT-PCR analysis of *Transglutaminase I*. Values are mean ± SE (n = 8). *P<0.05 (G) Effect of leptin on the migration of RT7 cells. Values are mean ± SE from two independent experiments performed in triplicate. *P<0.05, **P<0.01.

First, We Confirmed the Expression of *Ob-R* Mrna in RT7 Cells Using RT-PCR Analysis. This Examination Showed That RT7 Cells Expressed Mrna for *Ob-R* (Fig. 4A). to Elucidate the Effect of Leptin on the Proliferation of RT7 Cells, the Cells Were Cultured in the Absence Or Presence of Various Concentrations of Leptin. the Results Indicated That the Proliferation of RT7 Cells Was Not Affected by Leptin at Any Concentrations Examined (Fig. 4B). Next, the Effect of Leptin on the Differentiation/Function of RT7 Cells Was Investigated Using Semi-Quantitative RT-PCR Analysis of the Expression of Mrna Encoding Oral Mucosal Epithelial Cell-Related Genes, I.E. *Keratin 4*, *Keratin 10*, and *Transglutaminase I*. No Significant Effect of Leptin on the RT7 Cells Was Noted from This Analysis (Fig. 4C). to Evaluate and Quantitate Putative Existing Differences in Gene Expression, Quantitative RT-PCR Was Also Performed. This Analysis Reveled That the Gene Expression of *Keratin 10* and *Transglutaminase I* Was Slightly Suppressed by Leptin at a Concentration of 10 Ng/Ml on Day 7 of Culture (Fig. 4D–F). These Results Indicated That Leptin Exerted No Significant Effect on the Proliferation of RT7 Cells and That It Might Have a Modest Suppressive Effect on the Differentiation of RT7 Cells. Next, to Elucidate the Possible Effect of Leptin on the Migration of RT7 Cells, a Wound Healing Assay Was Performed. Interestingly, This Assay Revealed That Leptin Significantly Accelerated the Migration of RT7 Cells (Fig. 4G).

### Effect of leptin on expression of EGF and KGF in human oral mucosal epithelial cells

ELISA for EGF and KGF in supernatant of RT7 cells failed to determine the quantity, because these factors contained in supernatant was less than the lower detection limits (0.7 pg/ml for EGF and 15 pg/ml for KGF) even after 16-fold concentration (data not shown).

## Discussion

Oral mucosa is a primary and major barrier against physical, chemical and biological stimuli in the oral cavity, which acts as the entry point for the alimentary and respiratory tracts. Protection of this tissue is important not only for oral health but also for general health. However, oral mucosa is easily damaged by mechanical forces and surface abrasions caused by the normal activities involved in biting and chewing food. The oral mucosa is also easily affected by factors including systemic diseases/conditions such as diabetes, vascular disease and renal failure, malnutrition, smoking, infection, immunosuppression, and chemotherapy and radiotherapy for malignancies. In the presence of these factors, oral wounds often fail to heal adequately, resulting in chronic ulcer formation followed by serious systemic infections [31], [32]. It is important to establish an effective strategy to restore the structure and function of oral mucosa by promoting wound healing.

For many years, leptin-deficient *ob/ob* mice have been used as a model system to investigate the cellular and molecular mechanisms of impaired wound healing. The severe impairment of wound healing observed in these model mice was originally explained by the diabetic phenotype of the animals. However, a critical study by Frank *et al.* demonstrated that leptin markedly improved re-epithelialization of excisional skin wounds in *ob/ob* mice and even accelerated normal cutaneous wound healing in wild-type mice [19]. Recently, although leptin was reported to be present in saliva [27], its physiological role has yet to be fully elucidated. In this study, to elucidate the physiological role of leptin in oral cavity, we investigated the possibility that leptin may promote the wound healing of oral mucosa as well as skin.

As the first step, an immunohistochemical analysis for the expression of Ob-R protein in human and rabbit oral mucosa was performed. As a result, we found that Ob-R was expressed in the spinous/granular cells of epithelial tissue and the vascular endothelial cells in subepithelial connective tissue. Importantly, the expression pattern of Ob-R in oral mucosa was common between human and rabbit. These findings suggest that epithelial cells and vascular endothelial cells in oral mucosa are target cells for leptin.

Next, we investigated the effect of leptin on wound healing in the oral mucosa by using a rabbit model and a system of chemical wounds. These experiments showed that the wound area decreased much faster in the leptin-treated group compared with the control group. The time required for complete healing of the ulcerated area was also shortened in the leptin-treated group. BW, and levels of AST, ALT or BS were not significantly affected by the administration of leptin throughout the experimental period. These findings indicate that topically-administered leptin is capable of promoting wound healing of the oral mucosa without any systemic adverse effects. Histometric analysis of wounded gingival tissue showed that significantly more blood vessels were distributed in the connective tissue beneath the ulcer in the leptin-treated group compared with the control group. This finding strongly suggests that leptin stimulates angiogenesis in the connective tissue beneath the ulcer, and promotes wound healing in the oral mucosa by accelerating the supply of nutrients, oxygen and even some bioactive substances.

To investigate another possible mechanism underlying leptin’s promotive effect on oral mucosal wound healing, cell biological assays were performed using RT7 cells, a human oral mucosal epithelial cell line proven to express *Ob-R* by RT-PCR analysis. The cell proliferation assay using this cell line showed no stimulatory effects of leptin on the proliferation of RT7 cells at any concentrations examined. In addition, quantitative RT-PCR analysis was performed to examine whether leptin has any influence on the differentiation and/or function of oral mucosal epithelial cells. This analysis showed that 10 ng/ml of leptin slightly inhibited the expression of mRNA encoding *Keratin 10* or *Transglutaminase I* in RT7 cells. This result suggested that leptin might have a modest suppressive effect on the differentiation/function of RT7 cells. Next, to examine the effect of leptin on cell migration, a wound healing assay using RT7 cells was performed. This assay showed that leptin promoted the migration of RT7 cells. Given that the proliferation of these cells was not affected by leptin as mentioned above, we conclude that leptin is capable of stimulating cell migration of human oral mucosal epithelial cells.

Some previous studies reported that leptin induced the proliferation of various other cells such as skin keratinocytes [19], [21], lung epithelial cells [33], hemopoietic cells [34], pancreatic beta cells [35], [36], monocytes [37] and endothelial cells [17]. Contrary to these reports, the present study showed no stimulatory effects of leptin on the proliferation of RT7 cells. Unfortunately, it is impossible in this study to explain the reason/mechanism of this discrepancy. However, judging from the fact that our study is the first one on the effect of leptin on oral mucosal epithelial cells, one possibility is that oral mucosal epithelial cells might have distinctive characteristics different from other types of cells. This issue should be elucidated in the future investigation.

Wound healing in the oral mucosa is a complicated process involving the integration of multiple biological pathways. Several growth factors such as vascular endothelial growth factor [38], [39], fibroblast growth factor [40], [41] and the transforming growth factor β family [42], [43], [44] are known to act synergistically in the wound healing process. Therefore, it is difficult to rule out the possible involvement of these growth factors in the wound healing promoted by leptin. In fact, it has been demonstrated that leptin induces the expression of epidermal growth factor (EGF) and keratinocyte growth factor (KGF) in human oral keratinocytes [45]. Therefore, we investigated whether the synthesis of EGF and KGF in RT7 cells was affected by exogenous leptin at concentrations between 0.1 and 20 ng/ml using ELISA kits (R&D Systems, Inc., Minneapolis, USA). Unfortunately, however, these assays failed to determine the quantity because the amount of these factors contained in supernatant of RT7 cells was less than the lower detection limits (0.7 pg/ml for EGF and 15 pg/ml for KGF) even after 16-fold concentration. We were unable to clarify the reason for this discrepancy in the present study.

It is sure that saliva of rabbits contains some levels of leptin normally produced. From this fact, it would be important to figure out the endogenous levels of leptin in rabbit saliva. As for leptin concentrations in saliva, only two reports are currently available. In these reports, the concentration of leptin in human saliva is reported to be 0–3.5 ng/ml [27] or 6.19±2.1 ng/ml [28]. In contrast, there are no reports about leptin levels in rabbit saliva. Although we tried to determine the endogenous levels of leptin in rabbit saliva, it was impossible to collect saliva enough for analysis of leptin levels. This issue should be clearly settled in the future study.

The available evidence suggests several possibilities regarding the mechanisms by which leptin promotes wound healing. One possibility is that leptin promotes wound healing by enhancing the epithelial cell proliferation [20], [21]. Another possibility is that leptin promotes it by stimulating the angiogenesis [17], [18]. As the wound healing is a very dynamic and complex process, it is likely that these mechanisms contribute toward the wound healing concurrently. In the present study, we clearly demonstrated for the first time that leptin promotes wound healing in the oral mucosa by accelerating epithelial cell migration as well as enhancing angiogenesis around the wounded area. These results help explain the physiological function of leptin in saliva of promoting wound healing, and suggest a possible use of leptin in the treatment and/or prevention of abnormal conditions in oral cavity such as lichen planus and intractable stomatitis occurring as a result of radiotherapy and/or chemotherapy for head and neck cancers.
